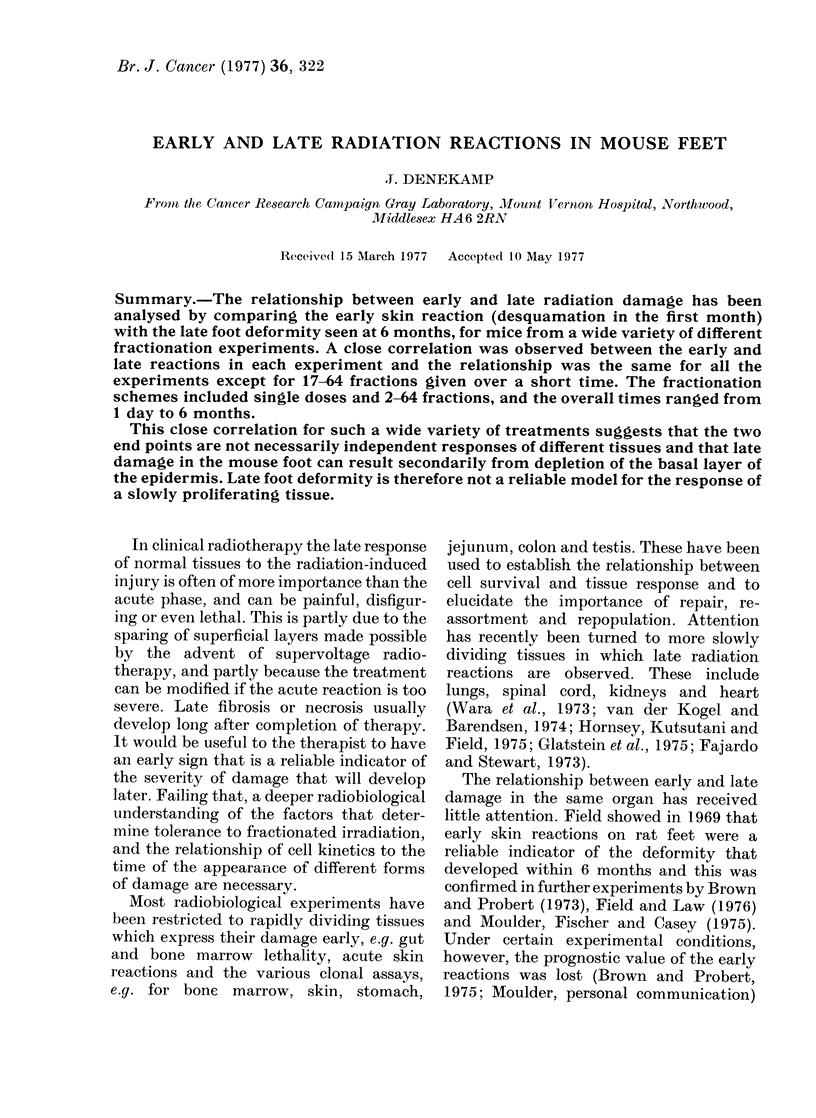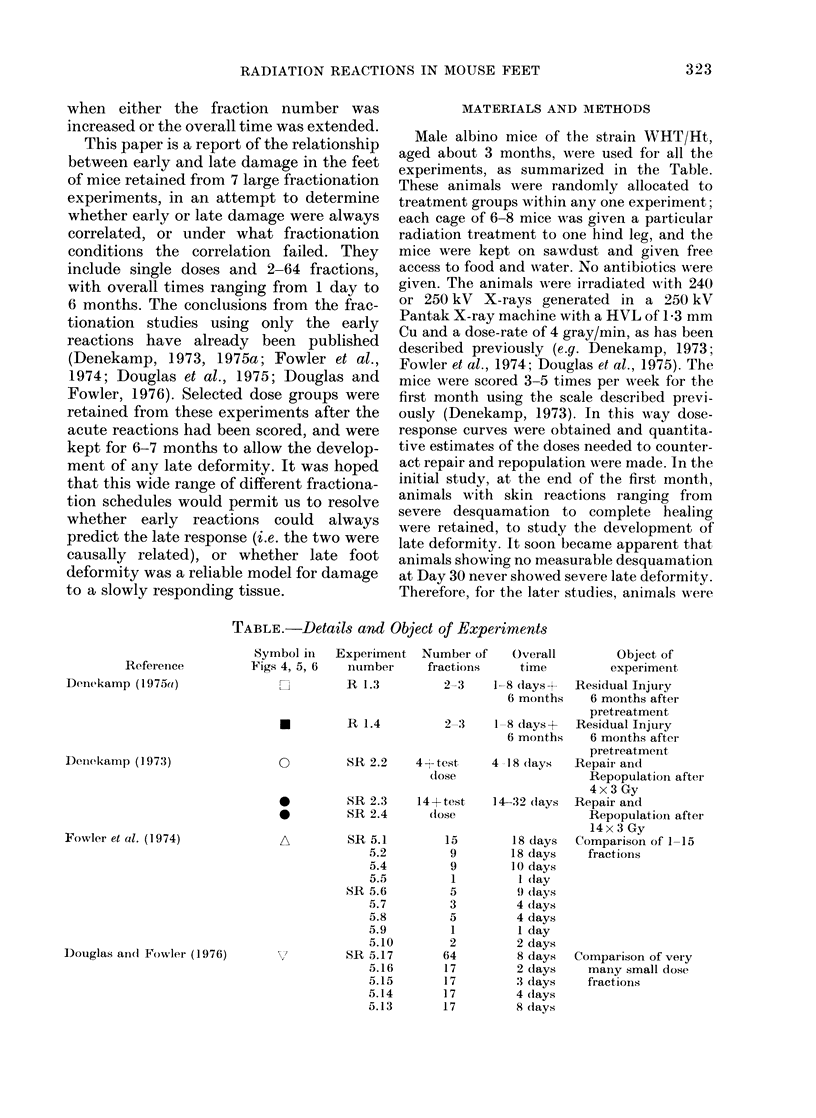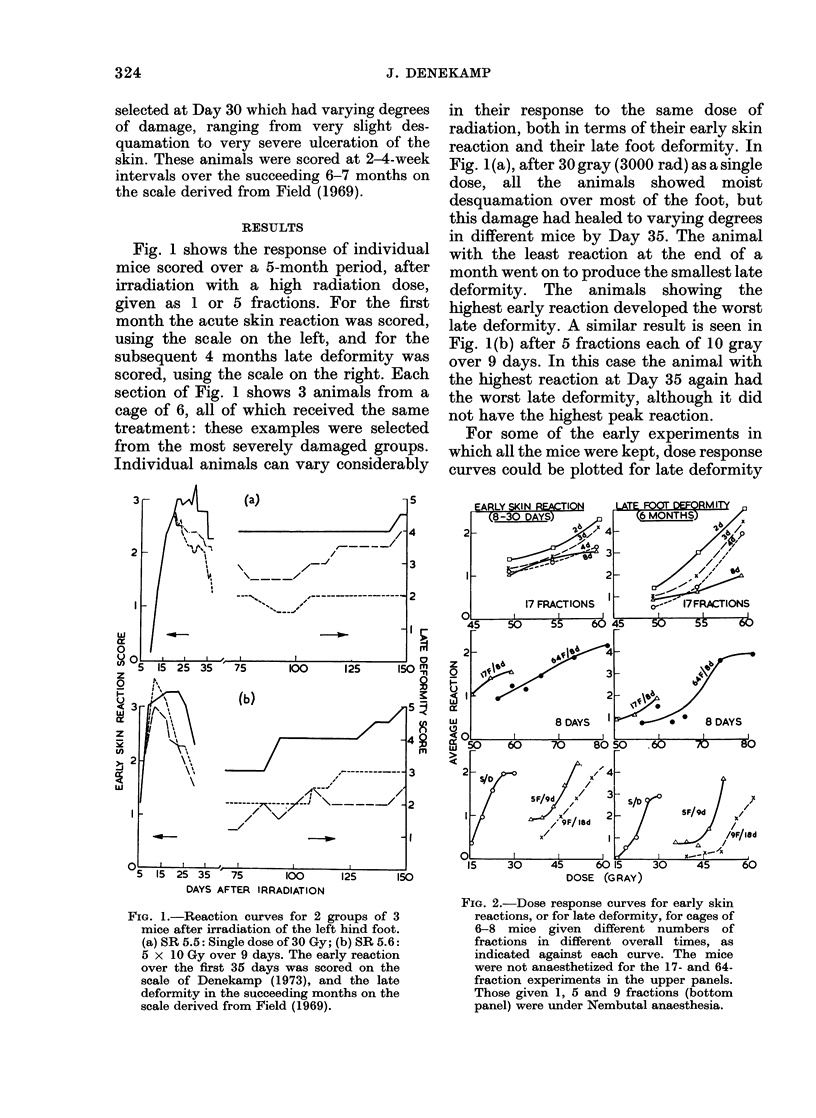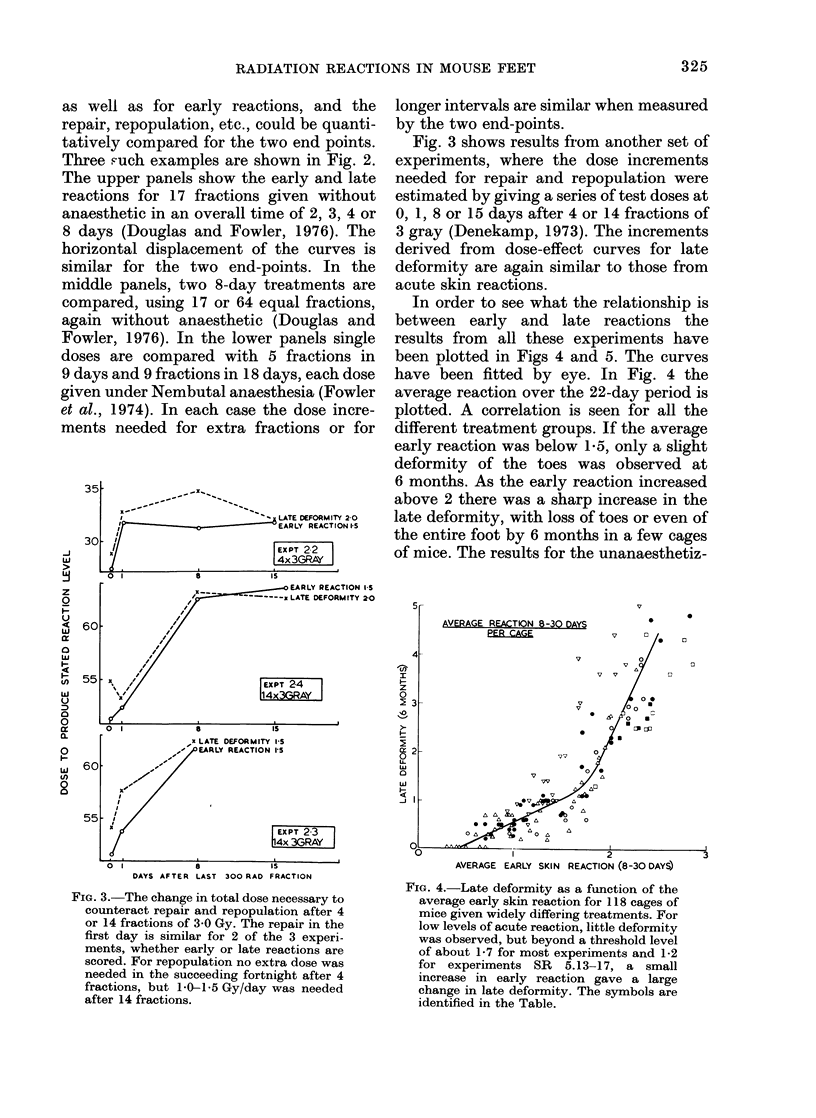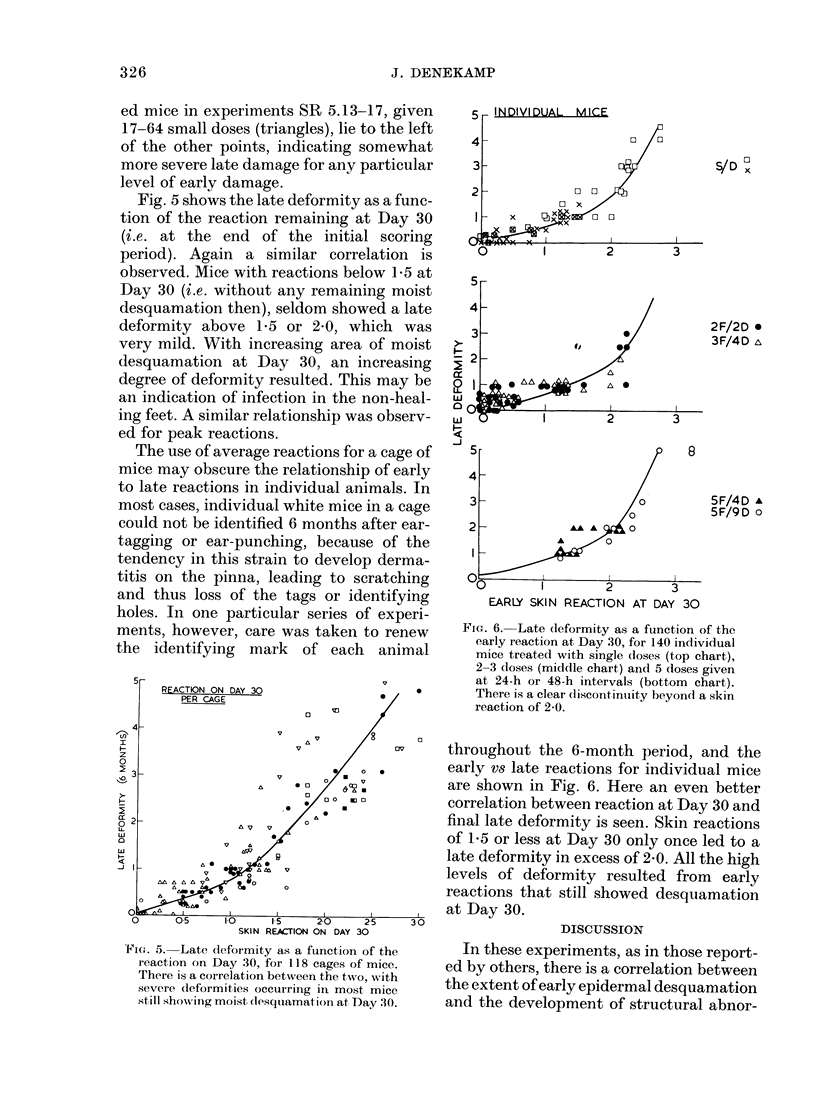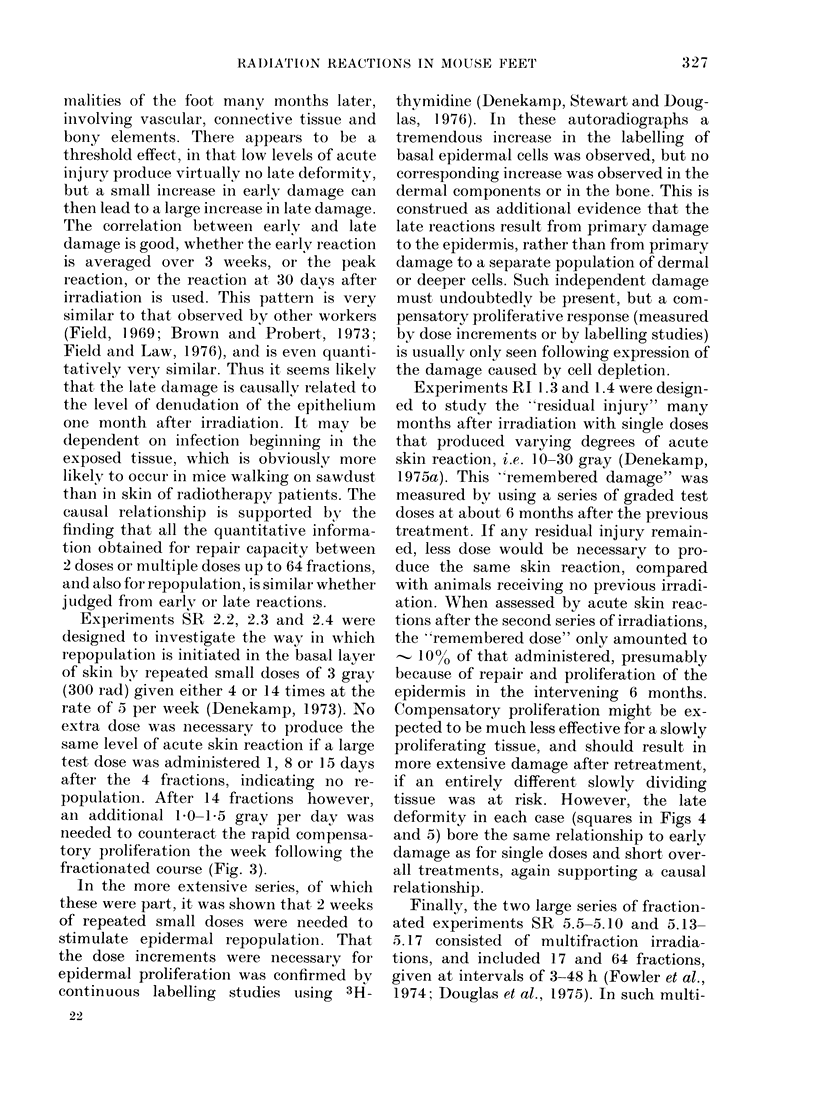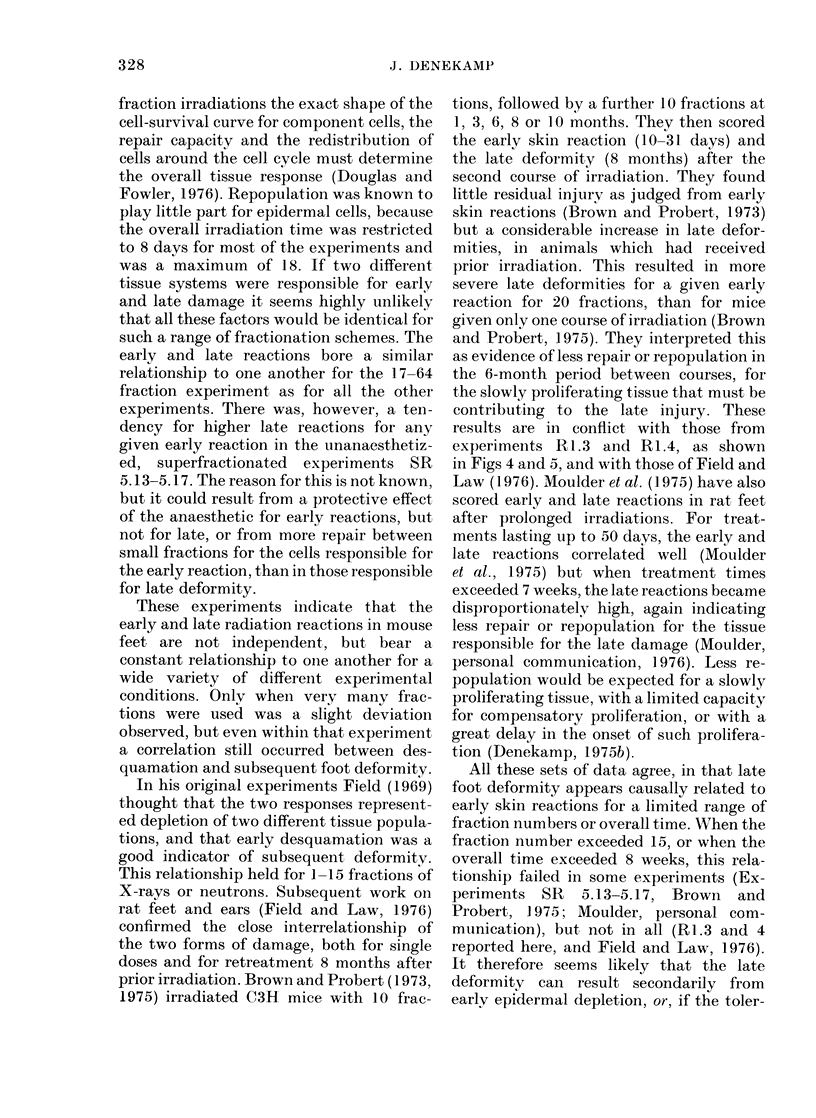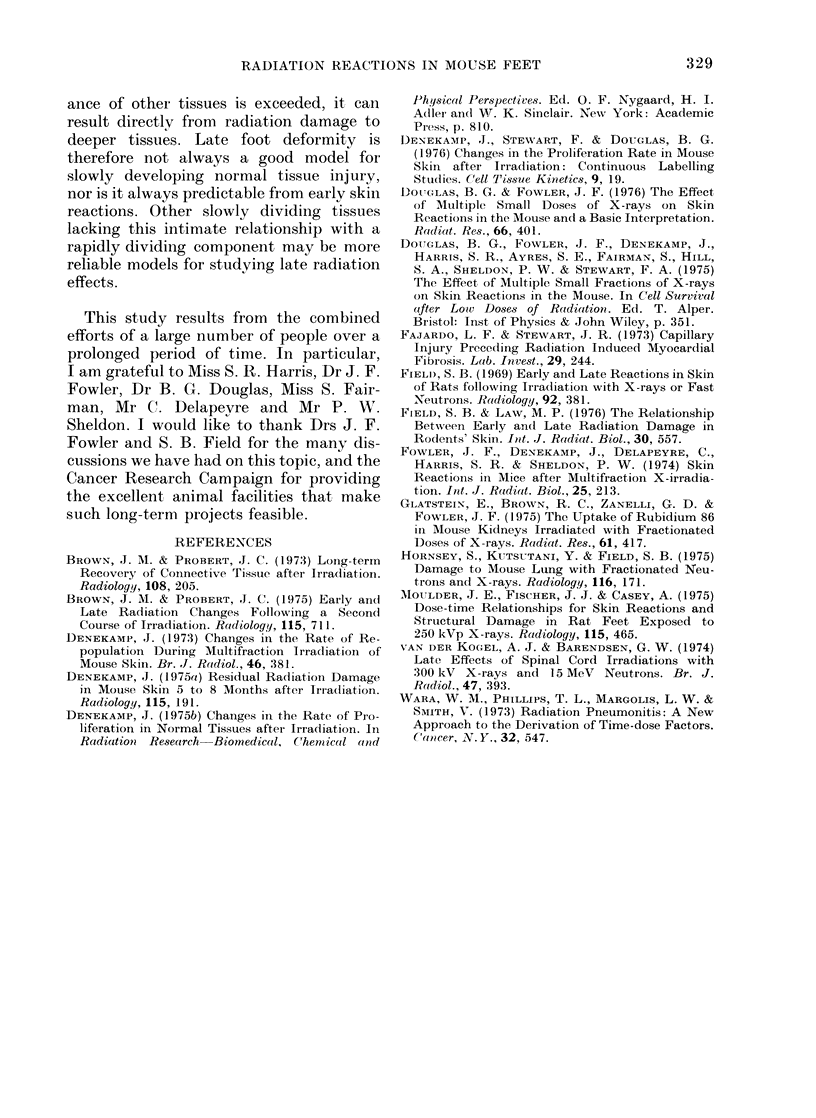# Early and late radiation reactions in mouse feet.

**DOI:** 10.1038/bjc.1977.196

**Published:** 1977-09

**Authors:** J. Denekamp

## Abstract

The relationship between early and late radiation damage has been analysed by comparing the early skin reaction (desquamation in the first month) with the late foot deformity seen at 6 months, for mice from a wide variety of different fractionation experiments. A close correlation was observed between the early and late reactions in each experiment and the relationship was the same for all the experiments except for 17-64 fractions given over a short time. The fractionation schemes included single doses and 2-64 fractions, and the overall times ranged from 1 day to 6 months. This close correlation for such a wide variety of treatments suggests that the two end points are not necessarily independent responses of different tissues and that late damage in the mouse foot can result secondarily from depletion of the basal layer of the epidermis. Late foot deformity is therefore not a reliable model for the response of a slowly proliferating tissue.


Br. J. Cancer (1977) 36, 322

### EARLY AND LATE RADIATION REACTIONS IN MOUSE FEET

J. DENEKAMP

*From the Cancer Research Campaign Gray Laboratory, Mount Vernon Hospital, Northwood, Middlesex HA6 2RN*

Received 15 March 1977 Accepted 10 May 1977

**Summary.—The relationship between early and late radiation damage has been analysed by comparing the early skin reaction (desquamation in the first month) with the late foot deformity seen at 6 months, for mice from a wide variety of different fractionation experiments. A close correlation was observed between the early and late reactions in each experiment and the relationship was the same for all the experiments except for 17–64 fractions given over a short time. The fractionation schemes included single doses and 2–64 fractions, and the overall times ranged from 1 day to 6 months.**

**This close correlation for such a wide variety of treatments suggests that the two end points are not necessarily independent responses of different tissues and that late damage in the mouse foot can result secondarily from depletion of the basal layer of the epidermis. Late foot deformity is therefore not a reliable model for the response of a slowly proliferating tissue.**

In clinical radiotherapy the late response of normal tissues to the radiation-induced injury is often of more importance than the acute phase, and can be painful, disfiguring or even lethal. This is partly due to the sparing of superficial layers made possible by the advent of supervoltage radiotherapy, and partly because the treatment can be modified if the acute reaction is too severe. Late fibrosis or necrosis usually develop long after completion of therapy. It would be useful to the therapist to have an early sign that is a reliable indicator of the severity of damage that will develop later. Failing that, a deeper radiobiological understanding of the factors that determine tolerance to fractionated irradiation, and the relationship of cell kinetics to the time of the appearance of different forms of damage are necessary.

Most radiobiological experiments have been restricted to rapidly dividing tissues which express their damage early, *e.g.* gut and bone marrow lethality, acute skin reactions and the various clonal assays, *e.g.* for bone marrow, skin, stomach, jejunum, colon and testis. These have been used to establish the relationship between cell survival and tissue response and to elucidate the importance of repair, reassortment and repopulation. Attention has recently been turned to more slowly dividing tissues in which late radiation reactions are observed. These include lungs, spinal cord, kidneys and heart (Wara *et al.*, 1973; van der Kogel and Barendsen, 1974; Hornsey, Kutsutani and Field, 1975; Glatstein *et al.*, 1975; Fajardo and Stewart, 1973).

The relationship between early and late damage in the same organ has received little attention. Field showed in 1969 that early skin reactions on rat feet were a reliable indicator of the deformity that developed within 6 months and this was confirmed in further experiments by Brown and Probert (1973), Field and Law (1976) and Moulder, Fischer and Casey (1975). Under certain experimental conditions, however, the prognostic value of the early reactions was lost (Brown and Probert, 1975; Moulder, personal communication)

when either the fraction number was increased or the overall time was extended.

This paper is a report of the relationship between early and late damage in the feet of mice retained from 7 large fractionation experiments, in an attempt to determine whether early or late damage were always correlated, or under what fractionation conditions the correlation failed. They include single doses and 2–64 fractions, with overall times ranging from 1 day to 6 months. The conclusions from the fractionation studies using only the early reactions have already been published (Denekamp, 1973, 1975*a*; Fowler *et al.*, 1974; Douglas *et al.*, 1975; Douglas and Fowler, 1976). Selected dose groups were retained from these experiments after the acute reactions had been scored, and were kept for 6–7 months to allow the development of any late deformity. It was hoped that this wide range of different fractionation schedules would permit us to resolve whether early reactions could always predict the late response (*i.e.* the two were causally related), or whether late foot deformity was a reliable model for damage to a slowly responding tissue.

### MATERIALS AND METHODS

Male albino mice of the strain WHT/Ht, aged about 3 months, were used for all the experiments, as summarized in the Table. These animals were randomly allocated to treatment groups within any one experiment; each cage of 6–8 mice was given a particular radiation treatment to one hind leg, and the mice were kept on sawdust and given free access to food and water. No antibiotics were given. The animals were irradiated with 240 or 250 kV X-rays generated in a 250 kV Pantak X-ray machine with a HVL of 1·3 mm Cu and a dose-rate of 4 gray/min, as has been described previously (*e.g.* Denekamp, 1973; Fowler *et al.*, 1974; Douglas *et al.*, 1975). The mice were scored 3–5 times per week for the first month using the scale described previously (Denekamp, 1973). In this way dose-response curves were obtained and quantitative estimates of the doses needed to counteract repair and repopulation were made. In the initial study, at the end of the first month, animals with skin reactions ranging from severe desquamation to complete healing were retained, to study the development of late deformity. It soon became apparent that animals showing no measurable desquamation at Day 30 never showed severe late deformity. Therefore, for the later studies, animals were

TABLE.—*Details and Object of Experiments*

| Reference | Symbol in Figs 4, 5, 6 | Experiment number | Number of fractions | Overall time | Object of experiment |
|---|---|---|---|---|---|
| Denekamp (1975*a*) | □ | R 1.3 | 2–3 | 1–8 days + 6 months | Residual Injury 6 months after pretreatment |
| | ■ | R 1.4 | 2–3 | 1–8 days + 6 months | Residual Injury 6 months after pretreatment |
| Denekamp (1973) | ○ | SR 2.2 | 4 + test dose | 4–18 days | Repair and Repopulation after 4 × 3 Gy |
| | ● | SR 2.3 | 14 + test dose | 14–32 days | Repair and Repopulation after 14 × 3 Gy |
| | ● | SR 2.4 | | | |
| Fowler *et al.* (1974) | △ | SR 5.1 | 15 | 18 days | Comparison of 1–15 fractions |
| | | 5.2 | 9 | 18 days | |
| | | 5.4 | 9 | 10 days | |
| | | 5.5 | 1 | 1 day | |
| | | SR 5.6 | 5 | 9 days | |
| | | 5.7 | 3 | 4 days | |
| | | 5.8 | 5 | 4 days | |
| | | 5.9 | 1 | 1 day | |
| | | 5.10 | 2 | 2 days | |
| Douglas and Fowler (1976) | ▽ | SR 5.17 | 64 | 8 days | Comparison of very many small dose fractions |
| | | 5.16 | 17 | 2 days | |
| | | 5.15 | 17 | 3 days | |
| | | 5.14 | 17 | 4 days | |
| | | 5.13 | 17 | 8 days | |

selected at Day 30 which had varying degrees of damage, ranging from very slight desquamation to very severe ulceration of the skin. These animals were scored at 2–4-week intervals over the succeeding 6–7 months on the scale derived from Field (1969).

### RESULTS

Fig. 1 shows the response of individual mice scored over a 5-month period, after irradiation with a high radiation dose, given as 1 or 5 fractions. For the first month the acute skin reaction was scored, using the scale on the left, and for the subsequent 4 months late deformity was scored, using the scale on the right. Each section of Fig. 1 shows 3 animals from a cage of 6, all of which received the same treatment: these examples were selected from the most severely damaged groups. Individual animals can vary considerably in their response to the same dose of radiation, both in terms of their early skin reaction and their late foot deformity. In Fig. 1(a), after 30 gray (3000 rad) as a single dose, all the animals showed moist desquamation over most of the foot, but this damage had healed to varying degrees in different mice by Day 35. The animal with the least reaction at the end of a month went on to produce the smallest late deformity. The animals showing the highest early reaction developed the worst late deformity. A similar result is seen in Fig. 1(b) after 5 fractions each of 10 gray over 9 days. In this case the animal with the highest reaction at Day 35 again had the worst late deformity, although it did not have the highest peak reaction.

For some of the early experiments in which all the mice were kept, dose response curves could be plotted for late deformity

Fig. 1.—Reaction curves for 2 groups of 3 mice after irradiation of the left hind foot. (a) SR 5.5: Single dose of 30 Gy; (b) SR 5.6: 5 × 10 Gy over 9 days. The early reaction over the first 35 days was scored on the scale of Denekamp (1973), and the late deformity in the succeeding months on the scale derived from Field (1969).

Fig. 2.—Dose response curves for early skin reactions, or for late deformity, for cages of 6–8 mice given different numbers of fractions in different overall times, as indicated against each curve. The mice were not anaesthetized for the 17- and 64-fraction experiments in the upper panels. Those given 1, 5 and 9 fractions (bottom panel) were under Nembutal anaesthesia.

as well as for early reactions, and the repair, repopulation, etc., could be quantitatively compared for the two end points. Three such examples are shown in Fig. 2. The upper panels show the early and late reactions for 17 fractions given without anaesthetic in an overall time of 2, 3, 4 or 8 days (Douglas and Fowler, 1976). The horizontal displacement of the curves is similar for the two end-points. In the middle panels, two 8-day treatments are compared, using 17 or 64 equal fractions, again without anaesthetic (Douglas and Fowler, 1976). In the lower panels single doses are compared with 5 fractions in 9 days and 9 fractions in 18 days, each dose given under Nembutal anaesthesia (Fowler *et al.*, 1974). In each case the dose increments needed for extra fractions or for

FIG. 3.—The change in total dose necessary to counteract repair and repopulation after 4 or 14 fractions of 3·0 Gy. The repair in the first day is similar for 2 of the 3 experiments, whether early or late reactions are scored. For repopulation no extra dose was needed in the succeeding fortnight after 4 fractions, but 1·0–1·5 Gy/day was needed after 14 fractions.

longer intervals are similar when measured by the two end-points.

Fig. 3 shows results from another set of experiments, where the dose increments needed for repair and repopulation were estimated by giving a series of test doses at 0, 1, 8 or 15 days after 4 or 14 fractions of 3 gray (Denekamp, 1973). The increments derived from dose-effect curves for late deformity are again similar to those from acute skin reactions.

In order to see what the relationship is between early and late reactions the results from all these experiments have been plotted in Figs 4 and 5. The curves have been fitted by eye. In Fig. 4 the average reaction over the 22-day period is plotted. A correlation is seen for all the different treatment groups. If the average early reaction was below 1·5, only a slight deformity of the toes was observed at 6 months. As the early reaction increased above 2 there was a sharp increase in the late deformity, with loss of toes or even of the entire foot by 6 months in a few cages of mice. The results for the unanaesthetiz-

FIG. 4.—Late deformity as a function of the average early skin reaction for 118 cages of mice given widely differing treatments. For low levels of acute reaction, little deformity was observed, but beyond a threshold level of about 1·7 for most experiments and 1·2 for experiments SR 5.13–17, a small increase in early reaction gave a large change in late deformity. The symbols are identified in the Table.

ed mice in experiments SR 5.13–17, given 17–64 small doses (triangles), lie to the left of the other points, indicating somewhat more severe late damage for any particular level of early damage.

Fig. 5 shows the late deformity as a function of the reaction remaining at Day 30 (*i.e.* at the end of the initial scoring period). Again a similar correlation is observed. Mice with reactions below 1·5 at Day 30 (*i.e.* without any remaining moist desquamation then), seldom showed a late deformity above 1·5 or 2·0, which was very mild. With increasing area of moist desquamation at Day 30, an increasing degree of deformity resulted. This may be an indication of infection in the non-healing feet. A similar relationship was observed for peak reactions.

The use of average reactions for a cage of mice may obscure the relationship of early to late reactions in individual animals. In most cases, individual white mice in a cage could not be identified 6 months after ear-tagging or ear-punching, because of the tendency in this strain to develop dermatitis on the pinna, leading to scratching and thus loss of the tags or identifying holes. In one particular series of experiments, however, care was taken to renew the identifying mark of each animal throughout the 6-month period, and the early *vs* late reactions for individual mice are shown in Fig. 6. Here an even better correlation between reaction at Day 30 and final late deformity is seen. Skin reactions of 1·5 or less at Day 30 only once led to a late deformity in excess of 2·0. All the high levels of deformity resulted from early reactions that still showed desquamation at Day 30.

Fig. 5.—Late deformity as a function of the reaction on Day 30, for 118 cages of mice. There is a correlation between the two, with severe deformities occurring in most mice still showing moist desquamation at Day 30.

Fig. 6.—Late deformity as a function of the early reaction at Day 30, for 140 individual mice treated with single doses (top chart), 2–3 doses (middle chart) and 5 doses given at 24-h or 48-h intervals (bottom chart). There is a clear discontinuity beyond a skin reaction of 2·0.

### DISCUSSION

In these experiments, as in those reported by others, there is a correlation between the extent of early epidermal desquamation and the development of structural abnormalities of the foot many months later, involving vascular, connective tissue and bony elements. There appears to be a threshold effect, in that low levels of acute injury produce virtually no late deformity, but a small increase in early damage can then lead to a large increase in late damage. The correlation between early and late damage is good, whether the early reaction is averaged over 3 weeks, or the peak reaction, or the reaction at 30 days after irradiation is used. This pattern is very similar to that observed by other workers (Field, 1969; Brown and Probert, 1973; Field and Law, 1976), and is even quantitatively very similar. Thus it seems likely that the late damage is causally related to the level of denudation of the epithelium one month after irradiation. It may be dependent on infection beginning in the exposed tissue, which is obviously more likely to occur in mice walking on sawdust than in skin of radiotherapy patients. The causal relationship is supported by the finding that all the quantitative information obtained for repair capacity between 2 doses or multiple doses up to 64 fractions, and also for repopulation, is similar whether judged from early or late reactions.

Experiments SR 2.2, 2.3 and 2.4 were designed to investigate the way in which repopulation is initiated in the basal layer of skin by repeated small doses of 3 gray (300 rad) given either 4 or 14 times at the rate of 5 per week (Denekamp, 1973). No extra dose was necessary to produce the same level of acute skin reaction if a large test dose was administered 1, 8 or 15 days after the 4 fractions, indicating no repopulation. After 14 fractions however, an additional 1·0–1·5 gray per day was needed to counteract the rapid compensatory proliferation the week following the fractionated course (Fig. 3).

In the more extensive series, of which these were part, it was shown that 2 weeks of repeated small doses were needed to stimulate epidermal repopulation. That the dose increments were necessary for epidermal proliferation was confirmed by continuous labelling studies using $^{3}$H-thymidine (Denekamp, Stewart and Douglas, 1976). In these autoradiographs a tremendous increase in the labelling of basal epidermal cells was observed, but no corresponding increase was observed in the dermal components or in the bone. This is construed as additional evidence that the late reactions result from primary damage to the epidermis, rather than from primary damage to a separate population of dermal or deeper cells. Such independent damage must undoubtedly be present, but a compensatory proliferative response (measured by dose increments or by labelling studies) is usually only seen following expression of the damage caused by cell depletion.

Experiments RI 1.3 and 1.4 were designed to study the "residual injury" many months after irradiation with single doses that produced varying degrees of acute skin reaction, *i.e.* 10–30 gray (Denekamp, 1975*a*). This "remembered damage" was measured by using a series of graded test doses at about 6 months after the previous treatment. If any residual injury remained, less dose would be necessary to produce the same skin reaction, compared with animals receiving no previous irradiation. When assessed by acute skin reactions after the second series of irradiations, the "remembered dose" only amounted to $\sim 10\%$ of that administered, presumably because of repair and proliferation of the epidermis in the intervening 6 months. Compensatory proliferation might be expected to be much less effective for a slowly proliferating tissue, and should result in more extensive damage after retreatment, if an entirely different slowly dividing tissue was at risk. However, the late deformity in each case (squares in Figs 4 and 5) bore the same relationship to early damage as for single doses and short overall treatments, again supporting a causal relationship.

Finally, the two large series of fractionated experiments SR 5.5–5.10 and 5.13–5.17 consisted of multifraction irradiations, and included 17 and 64 fractions, given at intervals of 3–48 h (Fowler *et al.*, 1974; Douglas *et al.*, 1975). In such multifraction irradiations the exact shape of the cell-survival curve for component cells, the repair capacity and the redistribution of cells around the cell cycle must determine the overall tissue response (Douglas and Fowler, 1976). Repopulation was known to play little part for epidermal cells, because the overall irradiation time was restricted to 8 days for most of the experiments and was a maximum of 18. If two different tissue systems were responsible for early and late damage it seems highly unlikely that all these factors would be identical for such a range of fractionation schemes. The early and late reactions bore a similar relationship to one another for the 17–64 fraction experiment as for all the other experiments. There was, however, a tendency for higher late reactions for any given early reaction in the unanaesthetized, superfractionated experiments SR 5.13–5.17. The reason for this is not known, but it could result from a protective effect of the anaesthetic for early reactions, but not for late, or from more repair between small fractions for the cells responsible for the early reaction, than in those responsible for late deformity.

These experiments indicate that the early and late radiation reactions in mouse feet are not independent, but bear a constant relationship to one another for a wide variety of different experimental conditions. Only when very many fractions were used was a slight deviation observed, but even within that experiment a correlation still occurred between desquamation and subsequent foot deformity.

In his original experiments Field (1969) thought that the two responses represented depletion of two different tissue populations, and that early desquamation was a good indicator of subsequent deformity. This relationship held for 1–15 fractions of X-rays or neutrons. Subsequent work on rat feet and ears (Field and Law, 1976) confirmed the close interrelationship of the two forms of damage, both for single doses and for retreatment 8 months after prior irradiation. Brown and Probert (1973, 1975) irradiated C3H mice with 10 fractions, followed by a further 10 fractions at 1, 3, 6, 8 or 10 months. They then scored the early skin reaction (10–31 days) and the late deformity (8 months) after the second course of irradiation. They found little residual injury as judged from early skin reactions (Brown and Probert, 1973) but a considerable increase in late deformities, in animals which had received prior irradiation. This resulted in more severe late deformities for a given early reaction for 20 fractions, than for mice given only one course of irradiation (Brown and Probert, 1975). They interpreted this as evidence of less repair or repopulation in the 6-month period between courses, for the slowly proliferating tissue that must be contributing to the late injury. These results are in conflict with those from experiments R1.3 and R1.4, as shown in Figs 4 and 5, and with those of Field and Law (1976). Moulder *et al.* (1975) have also scored early and late reactions in rat feet after prolonged irradiations. For treatments lasting up to 50 days, the early and late reactions correlated well (Moulder *et al.*, 1975) but when treatment times exceeded 7 weeks, the late reactions became disproportionately high, again indicating less repair or repopulation for the tissue responsible for the late damage (Moulder, personal communication, 1976). Less repopulation would be expected for a slowly proliferating tissue, with a limited capacity for compensatory proliferation, or with a great delay in the onset of such proliferation (Denekamp, 1975*b*).

All these sets of data agree, in that late foot deformity appears causally related to early skin reactions for a limited range of fraction numbers or overall time. When the fraction number exceeded 15, or when the overall time exceeded 8 weeks, this relationship failed in some experiments (Experiments SR 5.13–5.17, Brown and Probert, 1975; Moulder, personal communication), but not in all (R1.3 and 4 reported here, and Field and Law, 1976). It therefore seems likely that the late deformity can result secondarily from early epidermal depletion, *or*, if the tolerance of other tissues is exceeded, it can result directly from radiation damage to deeper tissues. Late foot deformity is therefore not always a good model for slowly developing normal tissue injury, nor is it always predictable from early skin reactions. Other slowly dividing tissues lacking this intimate relationship with a rapidly dividing component may be more reliable models for studying late radiation effects.

This study results from the combined efforts of a large number of people over a prolonged period of time. In particular, I am grateful to Miss S. R. Harris, Dr J. F. Fowler, Dr B. G. Douglas, Miss S. Fairman, Mr C. Delapeyre and Mr P. W. Sheldon. I would like to thank Drs J. F. Fowler and S. B. Field for the many discussions we have had on this topic, and the Cancer Research Campaign for providing the excellent animal facilities that make such long-term projects feasible.

### REFERENCES

Brown, J. M. & Probert, J. C. (1973) Long-term Recovery of Connective Tissue after Irradiation. *Radiology*, **108**, 205.

Brown, J. M. & Probert, J. C. (1975) Early and Late Radiation Changes Following a Second Course of Irradiation. *Radiology*, **115**, 711.

Denekamp, J. (1973) Changes in the Rate of Repopulation During Multifraction Irradiation of Mouse Skin. *Br. J. Radiol.*, **46**, 381.

Denekamp, J. (1975*a*) Residual Radiation Damage in Mouse Skin 5 to 8 Months after Irradiation. *Radiology*, **115**, 191.

Denekamp, J. (1975*b*) Changes in the Rate of Proliferation in Normal Tissues after Irradiation. In *Radiation Research—Biomedical, Chemical and Physical Perspectives*. Ed. O. F. Nygaard, H. I. Adler and W. K. Sinclair. New York: Academic Press, p. 810.

Denekamp, J., Stewart, F. & Douglas, B. G. (1976) Changes in the Proliferation Rate in Mouse Skin after Irradiation: Continuous Labelling Studies. *Cell Tissue Kinetics*, **9**, 19.

Douglas, B. G. & Fowler, J. F. (1976) The Effect of Multiple Small Doses of X-rays on Skin Reactions in the Mouse and a Basic Interpretation. *Radiat. Res.*, **66**, 401.

Douglas, B. G., Fowler, J. F., Denekamp, J., Harris, S. R., Ayres, S. E., Fairman, S., Hill, S. A., Sheldon, P. W. & Stewart, F. A. (1975) The Effect of Multiple Small Fractions of X-rays on Skin Reactions in the Mouse. In *Cell Survival after Low Doses of Radiation*. Ed. T. Alper. Bristol: Inst of Physics & John Wiley, p. 351.

Fajardo, L. F. & Stewart, J. R. (1973) Capillary Injury Preceding Radiation Induced Myocardial Fibrosis. *Lab. Invest.*, **29**, 244.

Field, S. B. (1969) Early and Late Reactions in Skin of Rats following Irradiation with X-rays or Fast Neutrons. *Radiology*, **92**, 381.

Field, S. B. & Law, M. P. (1976) The Relationship Between Early and Late Radiation Damage in Rodents' Skin. *Int. J. Radiat. Biol.*, **30**, 557.

Fowler, J. F., Denekamp, J., Delapeyre, C., Harris, S. R. & Sheldon, P. W. (1974) Skin Reactions in Mice after Multifraction X-irradiation. *Int. J. Radiat. Biol.*, **25**, 213.

Glatstein, E., Brown, R. C., Zanelli, G. D. & Fowler, J. F. (1975) The Uptake of Rubidium 86 in Mouse Kidneys Irradiated with Fractionated Doses of X-rays. *Radiat. Res.*, **61**, 417.

Hornsey, S., Kutsutani, Y. & Field, S. B. (1975) Damage to Mouse Lung with Fractionated Neutrons and X-rays. *Radiology*, **116**, 171.

Moulder, J. E., Fischer, J. J. & Casey, A. (1975) Dose-time Relationships for Skin Reactions and Structural Damage in Rat Feet Exposed to 250 kVp X-rays. *Radiology*, **115**, 465.

van der Kogel, A. J. & Barendsen, G. W. (1974) Late Effects of Spinal Cord Irradiations with 300 kV X-rays and 15 MeV Neutrons. *Br. J. Radiol.*, **47**, 393.

Wara, W. M., Phillips, T. L., Margolis, L. W. & Smith, V. (1973) Radiation Pneumonitis: A New Approach to the Derivation of Time-dose Factors. *Cancer, N.Y.*, **32**, 547.